# Methodology Using a Portable X-Ray Fluorescence Device for On-Site and Rapid Evaluation of Heavy-Atom Contamination in Wounds: A Model Study for Application to Plutonium Contamination

**DOI:** 10.1371/journal.pone.0101966

**Published:** 2014-07-10

**Authors:** Hiroshi Yoshii, Kouta Yanagihara, Hitoshi Imaseki, Tsuyoshi Hamano, Hirokuni Yamanishi, Masayo Inagaki, Yasuhiro Sakai, Nobuyuki Sugiura, Osamu Kurihara, Kazuo Sakai

**Affiliations:** 1 Research Center for Radiation Emergency Medicine, National Institute of Radiological Science, Chiba, Chiba, Japan; 2 Department of Physics, Faculty of Science, Toho University, Funabashi, Chiba, Japan; 3 Atomic Energy Research Institute, Kinki University, Higashi-Osaka, Osaka, Japan; 4 Radiation Environmental Effects Research Center, Nuclear Safety Research Association, Shinbashi, Minato, Tokyo, Japan; Kagoshima University Graduate School of Medical and Dental Sciences, Japan

## Abstract

Workers decommissioning the Fukushima-Daiichi nuclear power plant damaged from the Great East Japan Earthquake and resulting tsunami are at risk of injury with possible contamination from radioactive heavy atoms including actinides, such as plutonium. We propose a new methodology for on-site and rapid evaluation of heavy-atom contamination in wounds using a portable X-ray fluorescence (XRF) device. In the present study, stable lead was used as the model contaminant substitute for radioactive heavy atoms. First, the wound model was developed by placing a liquid blood phantom on an epoxy resin wound phantom contaminated with lead. Next, the correlation between the concentration of contaminant and the XRF peak intensity was formulated considering the thickness of blood exiting the wound. Methods to determine the minimum detection limit (MDL) of contaminants at any maximal equivalent dose to the wound by XRF measurement were also established. For example, in this system, at a maximal equivalent dose of 16.5 mSv to the wound and blood thickness of 0.5 mm, the MDL value for lead was 1.2 ppm (3.1 nmol). The radioactivity of ^239^Pu corresponding to 3.1 nmol is 1.7 kBq, which is lower than the radioactivity of ^239^Pu contaminating puncture wounds in previous severe accidents. In conclusion, the established methodology could be beneficial for future development of a method to evaluate plutonium contamination in wounds. Highlights: Methodology for evaluation of heavy-atom contamination in a wound was established. A portable X-ray fluorescence device enables on-site, rapid and direct evaluation. This method is expected to be used for evaluation of plutonium contamination in wounds.

## Introduction

The Fukushima-Daiichi nuclear power plant damaged from the Great East Japan Earthquake and resulting tsunami is currently being decommissioned. In the daunting task of decommissioning the plant, workers are at risk of injury with possible contamination from toxic radioactive heavy atoms including plutonium. Nuclear power plants or α-emitter handling facilities are equipped with survey meters for α-particle emissions [1]. Plutonium in the wound is, however, hard to detect because plutonium rarely emits γ-rays, and α-particles emitted from plutonium cannot pass through blood exiting the wound [2]. Genicot et al. [3] have developed a method for estimating the quantity of ^239^Pu from the intensity of weak 59.5 keV γ-rays emitted spontaneously from co-localized ^241^Am using a germanium semiconductor detector with calculations based on the known abundance ratio of ^239^Pu and ^241^Am. However, this passive detection method takes approximately 15 min. For more rapid diagnosis and initiation of the treatment of the wound, new methods for on-site and rapid evaluation of plutonium contamination in a wound are required.

To meet this requirement, we focused on X-ray fluorescence (XRF) analysis, which can be applied to elemental analysis. The intensity of the XRF peak depends on the number of corresponding atoms, and the number of atoms per unit radioactivity (i.e., per 1 Bq) is directly proportional to the half-life of the radionuclide. Thus, XRF analysis for a long-half-life radionuclide, such as plutonium, would be effective. Because XRF analysis is an active detection technique, the measurement time for XRF analysis is generally shorter than the passive detection of photons, X- and γ-rays, emitted spontaneously. Additionally, portable XRF devices can provide on-site measurement. Thus, XRF analysis using portable devices can potentially be used for on-site and rapid detection of plutonium-contaminated wounds.

XRF spectrometry has been used in the detection of contaminants in human tissue [4]–[9] and their phantoms [10]–[14]; however, it has not yet been applied to wounds. The first step in the application to wounds is to develop an XRF analysis methodology that considers the attenuation of X-rays by blood. The purpose of the present study is, therefore, to develop the methodology for on-site and rapid evaluation of contaminants in the wound using a portable XRF device considering the blood thickness. This study presents a methodology for evaluation of heavy-atom contamination in wounds using stable lead as the model contaminant, because lead is a familiar heavy atom and the XRF peak energies for Pb Lα (10.55 keV) and Pb Lβ (12.61 keV) are close to those for Pu Lα (14.28 keV) and Pu Lβ (18.28 keV), respectively [15]. In the absence of a standard wound model, we developed models for dry and bleeding wounds in this study. The contaminated wound phantom comprises an epoxy resin contaminated with stable lead, since the resin, with a density that is similar to that of human soft tissues, has been used previously to imitate the human skin [14]. The developed wound model has blood phantoms containing liquid blood combined with epoxy resin wound phantoms containing stable lead, for the XRF analysis, and developed the methodology to evaluate heavy-atom contamination in a wound considering the thickness of blood exiting the wound.

## Materials and Methods

### Bleeding wound model

**Figure 1:**
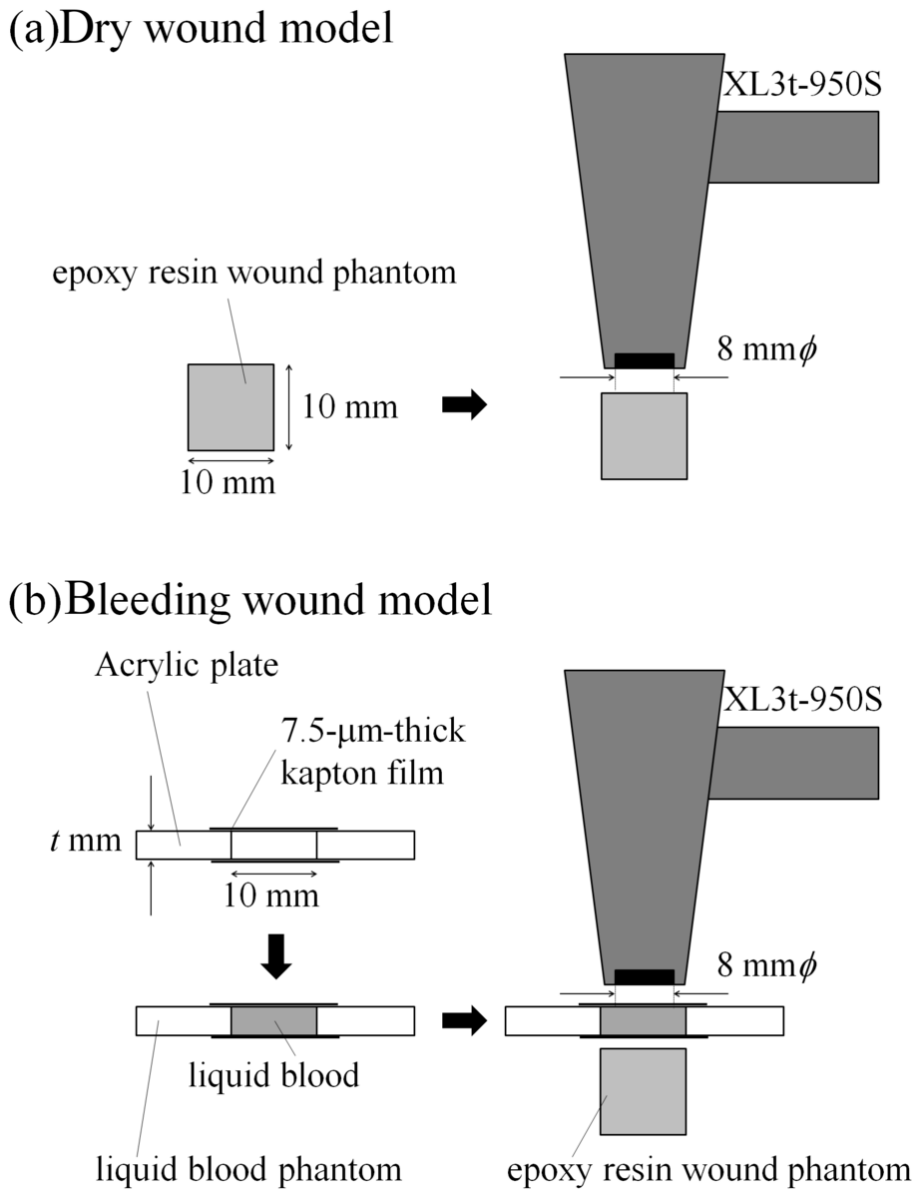
Schematic image of the measurement using a portable XRF device for dry and bleeding wound models.

The bleeding wound model with stable-lead contamination was constructed by placing the blood phantom on the epoxy resin wound phantom described below (Fig. 1b). The epoxy resin wound phantom without the blood phantom can be used as a dry wound model (Fig. 1a).

Epoxy resin wound phantoms were prepared by mixing epoxy resin (crystal resin, Nissin Resin Co., Ltd., Yokohama, Japan) with different concentrations of lead in white oil paint. In this study, lead white oil paint (Silver White, Kusakabe Co., Ltd., Saitama, Japan) containing 60.5% lead (from manufacture’s material safety data sheets) was used as the standard material. Solutions with different concentrations of lead (0, 200, 500, 1000, 1500 and 2000 ppm = µg/g) were prepared by precisely mixing appropriate volumes of the standard material and the solvent for oil paints (Odorless Petroleum, Holbein Works, Ltd., Osaka, Japan). Resulting solutions were mixed extensively with epoxy resin (paint solution-to-epoxy resin ratio = 1/99) to provide final lead concentrations of 0, 2, 5, 10, 15 and 20 ppm. Both the diameter and height of the molded epoxy resin wound phantoms were 10 mm (Fig. 1a). The density of the epoxy resin wound phantom was 1.06 g cm^–3^, which is similar to that of human soft tissue.

To investigate the effect of thickness of blood layer on the peak intensity of XRF, phantoms of blood with different thicknesses were constructed. For this, a circular piece (10-mm diameter) was cut out in each of the several acrylic plates (thickness = 0.5, 1.0, 1.5 and 2.0 mm). This 10-mm diameter opening could accommodate the irradiation field of the XRF device (8-mm diameter). One side of this circular aperture was sealed with a 7.5-µm-thick kapton film. Lead-free blood from mice (Japan SLC, Inc., Shizuoka, Japan) was then loaded into this opening. Finally, the opening on the other side was sealed by a second kapton film (Fig. 1b). Therefore, the constructed blood phantom had both sides of the aperture sealed by kapton films with a layer of blood enclosed in it. The specific gravity, which is a main factor affecting X-ray attenuation of mouse is similar to that of human blood [16], [17]. Furthermore, composition of elements identified by XRF in the region of this study, such as Fe, Zn, and Cu, are similar for both mouse and human blood [18]–[21]. Therefore, the attenuation of X-rays by mouse blood will be similar to that by human blood. In this study, lead was added to the epoxy resin, not to the blood. This was done to reflect the actual situation in radiation emergency. Typically, after an injury, the blood is washed off during the preliminary cleaning of the wound. Evaluation of contaminants adhering to a wound is performed subsequently, sometimes even while the fresh blood oozes out. To represent this situation, we constructed the bleeding wound model by placing the heavy metal-free blood phantom on the stable lead containing epoxy resin wound phantom. Thus, bleeding and dry wound models imitating wound with or without newly oozing blood were constructed.

### XRF apparatus

A portable XRF device, XL3t-950S (Thermo Fisher Scientific Inc.), which is easy to handle, was used in the present study. The silicon drift detector was installed in the XL3t-950S instead of the Si PIN diode used in the XL3 [8], and a silver (Ag) anode was employed. The diameter of the measurement range of the device is approximately 8 mm. In our measurements, the X-ray tube voltage was set to 50 kV, the current was set automatically, and the main filter of the XL3t-950S was selected. According to the XL3t-950S user’s manual, an equivalent dose to skin is less than 16.5 mSv for a 5 s exposure time (accumulation time) when the device is applied to the skin surface. In the present study, the maximal equivalent dose to the wound in a human was estimated from the accumulation time in the measurement using the bleeding wound model.

### Procedure

Accumulation times were set to 5, 10, 15 and 20 s. Because exposure dose to the wound is directly proportional to exposure time, the maximal equivalent doses to the wound are estimated as 16.5, 33.0, 49.5 and 66.0 mSv for the accumulation times of 5, 10, 15 and 20 s, respectively. XRF analysis was performed for 120 cases, the combination of six levels of lead concentration (*c*), five kinds of the blood phantom thickness (*t*) and four levels of maximal equivalent doses to the wound (*d*) estimated based on the accumulation time (*n* = 4, for each case). The measurement cases in the present study are indicated by these parameters; for example, (*c* = 2, *t* = 0.5, *d* = 16.5) indicates the measurement using the bleeding wound model constructed by the epoxy resin wound phantom containing 2-ppm stable lead (*c* = 2) and the blood phantom, whose thickness is 0.5 mm (*t* = 0.5), at a maximal equivalent dose to the wound estimated as 16.5 mSv (*d* = 16.5) obtained from the accumulation time of 5 s. Here, *t* = 0 mm means that no blood phantom was placed on the epoxy resin wound phantom at each *c* and *d*. As described above (Fig. 1a), this is the dry wound model. In the present study, a gross intensity of the measured XRF peak was obtained as the integrated intensity rather than the result of Gaussian fitting, since Pb Lα and Lβ peaks have asymmetric shapes due to the presence of unresolved subcomponent peaks (such as Lα_1_ and Lα_2_ for Lα peak and Lβ_1_, Lβ_2_, Lβ_3_ and Lβ_4_ for Lβ peak) [15], [22]. Background spectra are the spectra for the model using the epoxy resin wound phantom without lead, i.e., the spectra for the cases of *c* = 0 ppm at each *t* and *d*. Because there are five different blood thicknesses and four different maximal exposure doses to the wound, the spectra for 20 cases can be regarded as the background spectra, respectively. Here, the background intensity for a specific peak (e.g., Pb Lα peak) is defined as the gross intensity in the background spectrum at the energy range where the corresponding peak (Pb Lα peak) was found in the spectra for the model using the lead-contaminated epoxy resin wound phantom. The net intensity of the peak was calculated after subtracting the background intensity from gross intensity.

## Results

### The measured spectra

**Figure 2:**
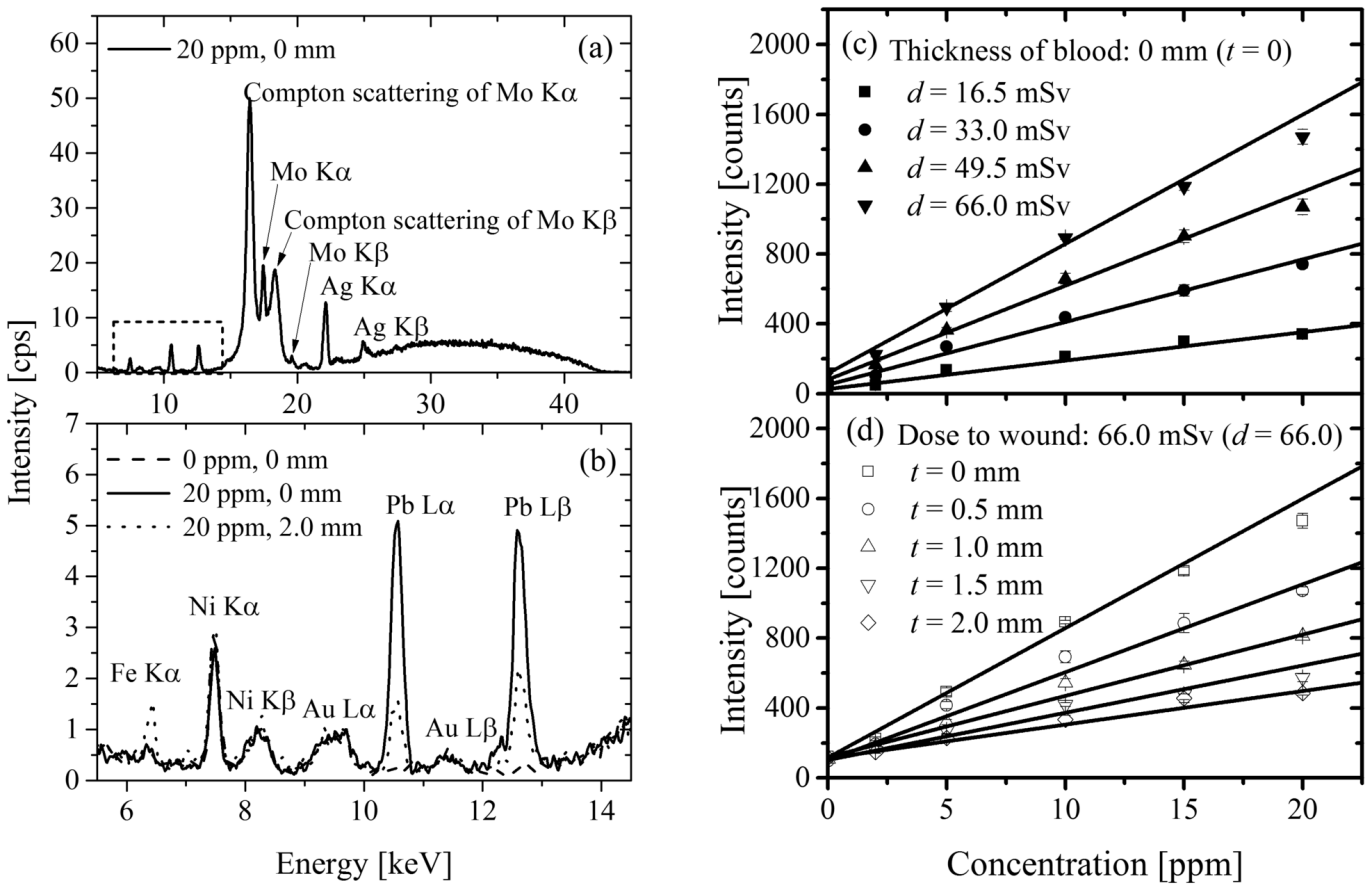
XRF spectra and the correlation between the concentration of lead and peak intensity. The panels on the left represent (a) representative XRF spectrum, (b) partial XRF spectra displaying Pb Lα and Pb Lβ peaks. The panels on the right represent the correlation between the concentration of lead in the epoxy resin wound phantom and (c) peak intensity for each maximal equivalent dose or (d) for each blood thickness (*n* = 4, for each data point). The error bars represent the standard deviations of the measurements.

A representative XRF spectrum is shown in Fig. 2a for the case of (*c* = 20, *t* = 0, *d* = 66.0). Device-derived peaks, including Ni Kα, Ni Kβ, Mo Kα and its Compton scattering, Mo Kβ and its Compton scattering, Ag Kα and Ag Kβ, were found in the measured spectrum. Since the anode of X-ray tube is silver, the peaks attributed to silver were clearly observed. The intense peaks of molybdenum were also observed, since molybdenum was present in the path of incident X-ray in the device. Fig. 2b is an enlarged view of the measured spectrum in the area enclosed by the dotted rectangle in Fig. 2a. Fig. 2b shows the spectra for the cases of (*c* = 0, *t* = 0, *d* = 66.0), (*c* = 20, *t* = 0, *d* = 66.0) and (*c* = 20, *t* = 2.0, *d* = 66.0). Here, the spectrum for the case of (*c* = 0, *t* = 0, *d* = 66.0) is the background spectrum for the dry wound model (*t* = 0) at *d* = 66.0 mSv. The cases of (*c* = 20, *t* = 0, *d* = 66.0) and (*c* = 20, *t* = 2.0, *d* = 66.0) are the dry and bleeding (at *t* = 2.0 mm) wound models using the epoxy resin wound phantom containing 20 ppm of lead, respectively. In the spectrum for the case of (*c* = 20, *t* = 0, *d* = 66.0), the peaks of Pb Lα (10.55 keV) and Pb Lβ (12.61 keV) can be clearly observed, though the background spectrum, i.e., the case of (*c* = 0, *t* = 0, *d* = 66.0), shows no clear peak at the corresponding energies. The background intensity at the energy of the Pb Lβ peak is larger than that at the energy of the Pb Lα peak due to the Compton scattering tail of Mo Kα. The Pb Lα peak rather than the Pb Lβ peak was, therefore, used to evaluate lead contamination in the present study. As found by comparison of the spectra for the cases of (*c* = 20, *t* = 0, *d* = 66.0) and (*c* = 20, *t* = 2.0, *d* = 66.0), intensities for both the Pb Lα and Pb Lβ peaks are reduced by putting the blood phantom on the epoxy resin wound phantom (Fig. 2b). The intensities of device-derived peaks for the case of (*c* = 20, *t* = 2.0, *d* = 66.0) are similar to those for the case of (*c* = 0, *t* = 0, *d* = 66.0). This indicates that the intensities for the device-derived peaks were not dependent on the blood thickness. Similarly, the background intensities at the Pb Lα peak for each blood thickness were the same within the margin of error (spectra not shown).

### Correlations between intensity of the XRF peak and lead concentration, dose to wound, and blood thickness

The gross intensities of the Pb Lα peak in the spectra for the dry wound model were plotted against lead concentration in the epoxy resin wound phantom for each maximal equivalent dose to the wound (Fig. 2c). In Fig. 2c, the background intensities at the Pb Lα peak for each maximal exposure dose to the wound are given as the intercept. Fig. 2c shows that the background intensities at the Pb Lα peak are directly proportional to the maximal equivalent dose to the wound (*d*). Since both the maximal equivalent dose to the wound and background intensity are directly proportional to the measurement time independently, it was reasonable to assume a direct relationship between background intensity and the maximal equivalent dose to the wound. The net intensity of the Pb Lα peak can be obtained by subtraction of the background intensity shown as the intercept in Fig. 2c from the gross intensity of the Pb Lα peak. The net intensity of the Pb Lα peak was directly proportional to both the lead concentration in the epoxy resin wound phantom (*c*) and the maximal equivalent dose to the wound (*d*). Generally, the net intensity of the XRF peak is directly proportional to the concentration of the target element and the accumulation time [22]. Since the maximal equivalent dose to the wound is directly proportional to the accumulation time, it is gratifying that the net intensity is directly proportional to *c* and *d*.

The curves of the gross intensity of the Pb Lα peak versus lead concentration in the epoxy resin wound phantom for each blood thickness at *d* = 66.0 mSv are drawn in Fig. 2d. The gross intensity of the Pb Lα peak was inversely correlated to the blood thickness for each concentration of lead in the epoxy resin wound phantom. There are no differences among the background intensities of the Pb Lα peak given as the intercept for several blood thicknesses.

## Discussion

In the past three decades, XRF has been used in the analysis of human tissue and their phantoms [4]–[14]. For example, Nie et al. developed a method to measure lead contamination in bone using a portable XRF spectrometer for the evaluation of human chronic exposure to lead [8]. However, XRF has not yet been used in the analysis of wounds. In this study, we described a portable XRF device-based method for on-site and rapid evaluation of heavy-atom contamination in a wound.

As shown in Fig. 2c, the background intensity at the Pb Lα peak is directly proportional to *d* and can be represented by *I*_0_ *d*, where the proportionality constant, *I*_0_, is the background intensity when *d* = 1 mSv. Similarly, the net intensity of the Pb Lα peak is directly proportional to both *d* and *c* and is represented by *I*_1_ *d c*, where the proportionality constant, *I*_1_, is the net intensity of the Pb Lα peak when *d* = 1 mSv and *c = *1 ppm. Because the gross intensity of the Pb Lα peak for the dry wound model *I*(*c*, *t* = 0, *d*) is the sum of the net intensity of the Pb Lα peak and background intensity, it can be expressed as
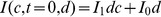
(1)


**Figure 3:**
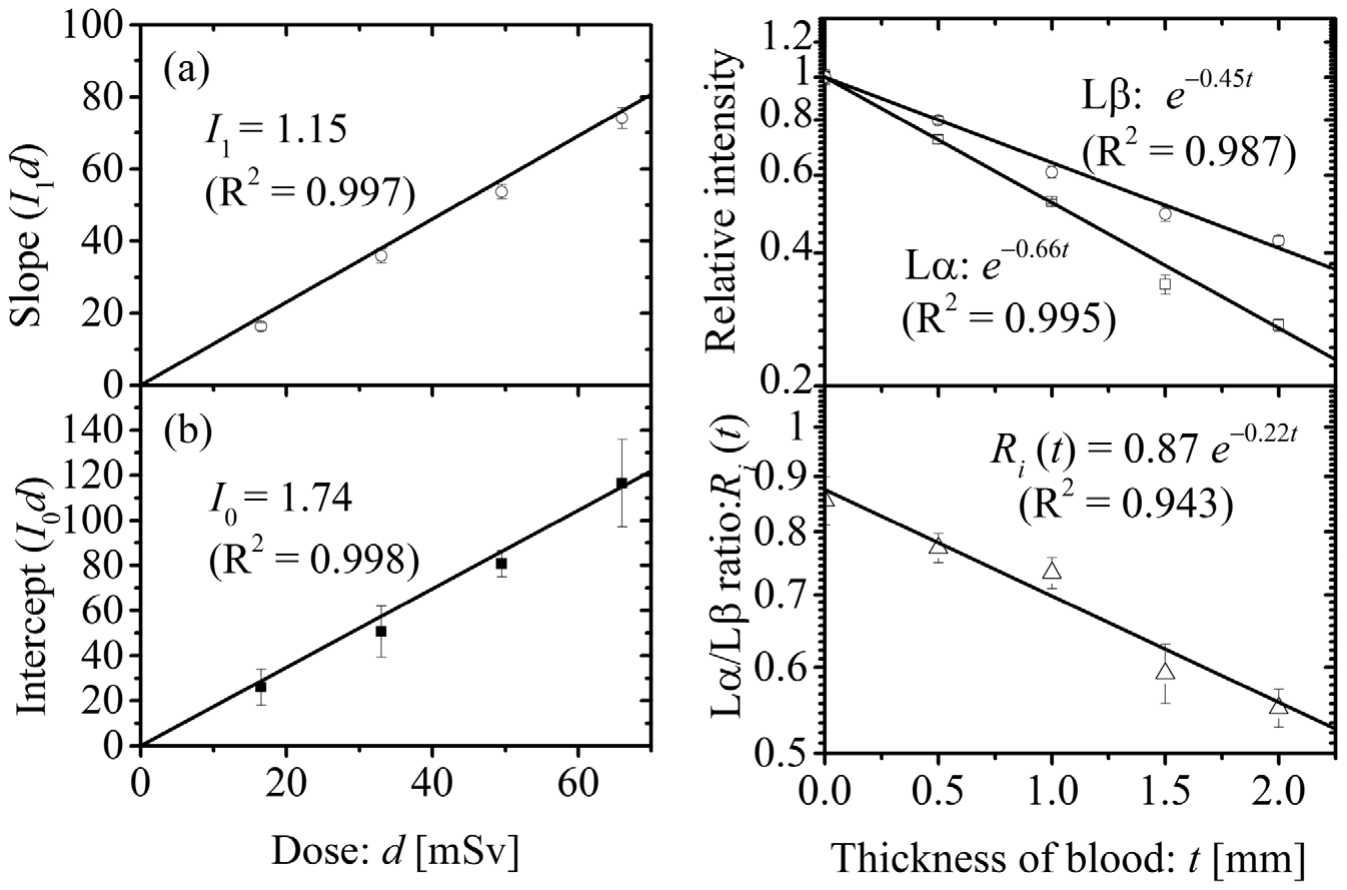
Determination of parameters. The correlations between maximal equivalent dose and (a) slope or (b) intercept (from Fig. 2c). Panels (c) and (d) represent the correlation between blood thickness and the (c) relative intensity or (d) Lα/Lβ ratio. The error bars represent the standard deviations of the measurements.



According to the equation (1), both the slope and the intercept shown in Fig. 2c, *I*_1_ *d* and *I*_0_ *d*, are directly proportional to *d* (Fig. 3a and 3b). The parameters *I*_1_ and *I*_0_ can be, therefore, obtained as the slope of the datasets in Fig. 3a and 3b, respectively. Next, we focused on the correlation between the intensity of the XRF peak and blood thickness. Generally, the measured intensity, *i*(*t*), transmitted through a layer of material with thickness, *t*, is related to the incident intensity, *i*_0_, according to the Lambert law:
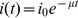
(2)where µ is called a linear attenuation coefficient. Because the net intensity, the first term in equation (1), obeys the Lambert law, the gross intensity of the Pb Lα peak for the bleeding wound model *I*(*c*, *t*, *d*) is expressed as

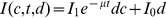
(3)The correlations between blood thickness and the net intensities of the Pb Lα and Pb Lβ peaks relative to those for the dry wound model are shown in Fig. 3c. The linear attenuation coefficients of liquid blood at the energies of the Pb Lα and Pb Lβ peaks can be obtained as the slopes of the datasets in Fig. 3c. As shown in Fig. 3c, the linear attenuation coefficient for the Pb Lα peak is larger than that for the Pb Lβ peak. The ratios of the net intensities for the Pb Lα peak to those for the Pb Lβ peak, Lα/Lβ ratio, are plotted against the blood thickness (Fig. 3d). The data of the Lα/Lβ ratio fit the straight line well in the semi-logarithmic plot, and this means the blood thickness, *t*, can be obtained from the observed Lα/Lβ ratio. Using *I*_1_, *I*_0_ and *µ* values for the Pb Lα peak obtained by least-square fittings (Fig. 3a, 3b and 3c), equation (3) can be rewritten as
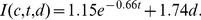
(4)


Here, blood thickness, *t*, can be obtained from the observed Lα/Lβ ratio as described above. By least-square fitting the data in Fig. 3d, the Lα/Lβ ratio, *R*_i_(*t*), is expressed as
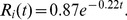
(5)


Thus, the blood thickness can be determined by applying the obtained Lα/Lβ ratio from the measured XRF spectrum to equation (5).

Based on our findings, the following method for evaluating lead contamination in a wound is proposed. First, the XRF measurement is performed under the monitoring intensities of the Pb Lα and Pb Lβ peaks. Second, the blood thickness is estimated by the Lα/Lβ ratio obtained from the measured XRF spectrum and equation (5). Finally, the obtained blood thickness and maximal equivalent dose to the wound obtained from the accumulation time are assigned to equation (4) and the concentration of lead contamination in the wound can be evaluated.

Next, we focus on the minimum detection limit (MDL) of the measurement. Generally, MDL is defined as the concentration that gives a signal three times the standard deviation of the background signal. The MDL value can be, therefore, defined as the concentration that gives the net intensity, *I*_1_ *e* ^−*µt*^ *d c*, three times the standard deviation of the background intensity, which is expressed as 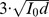, because the standard deviation of the background intensity is the square root of the background intensity. This value, 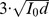, equals the net intensity when *c* = MDL as
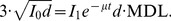
(6)


The MDL value is, therefore, expressed as
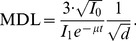
(7)


**Figure 4:**
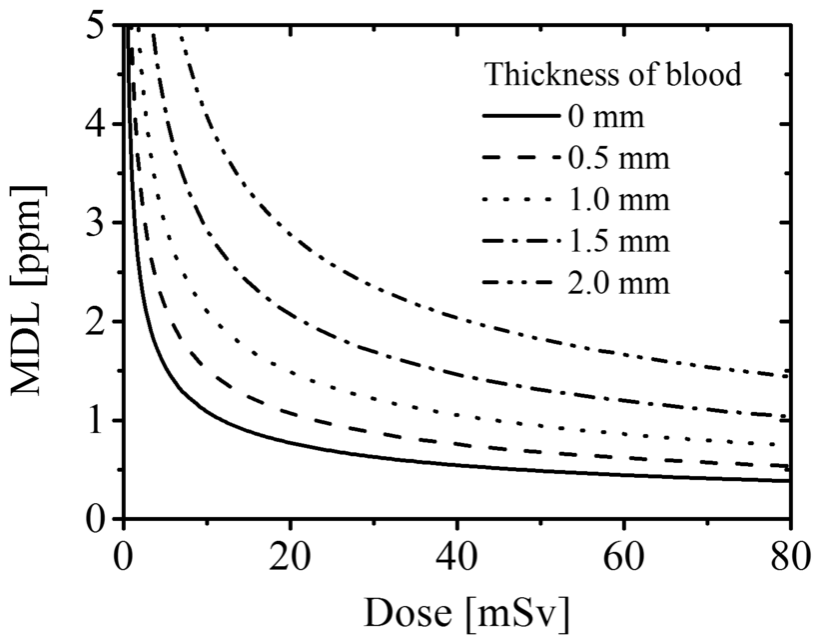
The correlation between maximal equivalent dose and MDL for each blood thickness.

As shown in equation (7), MDL is inversely proportional to the square root of the maximal equivalent dose to the wound. Fig. 4 shows the relation between maximal equivalent dose to the wound, *d*, and MDL (*d*-MDL curve) for each blood thickness (*t* = 0, 0.5, 1.0, 1.5 and 2.0 mm) using experimentally defined parameters, *I*_1_ (see Fig. 3a), *I*_0_ (see Fig. 3b) and µ (see Fig. 3c). The *d*-MDL curve for any blood thickness can be also drawn. According to the *d*-MDL curve, MDL at any maximal equivalent dose to the wound can be calculated. In the present study, four blood phantoms with thicknesses of *t* = 0.5, 1.0, 1.5 and 2.0, were prepared to consider the effect of blood thickness on the intensities of the signal. However, emergency first aid is given priority for patients with significant hemorrhage over evaluation of wound contaminants [23]. Thus, this method will be used on wounds with little hemorrhage, i.e., in which the blood exiting the wound is thin. For example, if the thickness of blood is 0.5 mm and the maximal equivalent dose to the wound is 16.5 mSv, which corresponds to 5 s of accumulation time, MDL for lead can be calculated as approximately 1.2 ppm. Considering the diameter of the measurement range for the portable XRF device used, the amount of substance in the measured region of the epoxy resin wound phantom containing 1.2 ppm lead is approximately 3.1 nmol. The corresponding radioactivity for the same amount of ^239^Pu is approximately 1.7 kBq, which is lower than the radioactivity of ^239^Pu contaminating puncture wounds in previous severe accidents [24], [25]. Therefore, this methodology could be applied to evaluate plutonium contamination in wounds and further tests on the feasibility are warranted. In the situation discussed above, the maximal equivalent dose to the area of a wound within the measurement field of the XRF device (diameter is 8 mm) is estimated to be 16.5 mSv. In a previous *in vivo* XRF measurement of patients [8], the equivalent dose to 1-cm^2^ area of skin was estimated as 13–26.5 mSv, which is comparable to the maximal equivalent dose to the wound estimated in the present study. Incidentally, the equivalent dose limit of human skin is recommended as 500 mSv annually for radiation workers by the International Commission on Radiological Protection (ICRP) [26]. Thus, the exposure dose in this method is acceptable.

For the evaluation of plutonium contamination in actual wounds in humans, the following modifications to the procedure used in the evaluation of lead contamination described above are recommended. Primarily, the treatment to stop the bleeding from the wound is carried out first. To examine the plutonium contamination in wound, materials collected during the initial treatment, which include blood absorbed in the swab, are dried and subjected to a preliminary test by α-survey meter. Although α-survey meter determines the total amount of α-particles emitted from several α-emitters, the presence of plutonium is strongly indicated when α-particles are counted. Under these circumstances, direct XRF measurement against the wound as described in this study is performed. For this analysis, the measuring time is initially adjusted to the maximum allowable time, which is calculated from the permissible radiation dose decided by clinician after considering the severity of the incident, predicted by the preliminary analysis. In a practical sense, establishment of guidelines for clinicians, which encourages rapid decision of measurement time, would be required. If there is concern for surface contamination on the skin surrounding the wound, masking the wound by a thin metallic sheet would be recommended. XRF analysis of the wound is then performed by monitoring the intensities of the Pu Lα and Lβ peaks. When the intensities of the peaks reach detectable levels for accurate determination of the concentration of plutonium, the measurement is stopped to ensure reduced radiation exposure. Finally, plutonium contamination in wound is quantified using equations (4) and (5), parameters for which are obtained from the measurement using the phantoms containing plutonium as described above. Such an accurate quantification can guide the subsequently employed therapeutic strategies. This method enables a patient to receive rapid treatment against contaminants (e.g., chelation therapy), which can reduce the internal exposure, although some additional skin doses would be given. Thus, the method described in this report can promote the use of the portable XRF device for real life emergencies.

This method rapidly evaluates the total plutonium content, but not the relative abundance of each isotope, which include ^239^Pu, ^240^Pu, and ^241^Pu. When an actual accident occurs in the nuclear reactor, the relative amounts of the isotopes in the nuclear fuel splattered from reactor can be immediately estimated from the entries in the operating log. Using the total amount of plutonium contamination in the wound obtained by the described analysis and the estimated isotopic distribution derived from the operating log, the amount of each plutonium isotope in the wound can be calculated. Such an estimate is beneficial for the treatment of plutonium contamination. In addition, the wound can be contaminated with other radioactive elements (e.g., uranium) and fission products (e.g.,^ 90^Sr, ^131^I, ^134^Cs, and ^137^Cs). However, these elements do not affect the background signal of the XRF peak for plutonium, since the XRF peak energy for plutonium is distinct and devoid of any overlap from the peaks attributed to those elements [15].

The XRF peak energies of Pu Lα (14.28 leV) and Pu Lβ (18.28 keV) are located in the region where intense background due to molybdenum peaks are observed. Therefore, we are developing a new device that uses only trace amounts of molybdenum near the path of incident X-ray. Additionally, to use this device for the analysis of a larger wound, our efforts are directed toward broadening the measurement range.

In this study, the epoxy resin wound phantoms constructed are 10-mm deep with a uniform distribution of contaminants to promote a conservative evaluation, which is accepted in radiation protection. A conservative evaluation is promoted for the following reason. In actual wounds, contaminants are considered to be usually located near the top of the wound. Since attenuation of X-rays increases with depth, the XRF intensity when contaminants are distributed at the surface of the wound is higher than that when the same amount of contaminant is uniformly distributed toward a 10 mm depth. Therefore, the amount of contaminants evaluated by the calibration curve obtained using a 10-mm-depth wound phantom is higher than the true value. Thus, the proposed method gives a conservative evaluation. Additionally, since the intensity of the XRF peak depends on the total amount of target atom, the quantitative evaluation of the plutonium contamination in wound can be determined even when the distribution of plutonium is laterally heterogeneous.

In conclusion, a methodology using a portable XRF device for on-site, rapid and direct evaluation of heavy-atom contamination in a wound was established using a bleeding wound model comprising an epoxy resin wound phantom containing contaminants and a blood phantom containing liquid blood. Developing the methodology using the liquid blood phantom was performed for the first time. The results of the present study indicate this methodology is applicable to evaluate plutonium contamination, which could contribute to the advancement of crisis management in the decommissioning of the Fukushima-Daiichi nuclear power plant. This method can also to be used for the analysis for contamination by actinides for injuries that occur in actinide handling facilities.
